# Towards IoT-Fog-ML integration for temperature break detection and prediction in fresh produce cold chains: a systematic review and architectural framework

**DOI:** 10.3389/frai.2026.1830032

**Published:** 2026-07-17

**Authors:** Jeremiah Taguta, Jean Frederic Isingizwe Nturambirwe, Clement Nthambazale Nyirenda

**Affiliations:** 1 Department of Computer Science, University of the Western Cape, Cape Town, Western Cape, South Africa; 2 eResearch Office, University of the Western Cape, Cape Town, Western Cape, South Africa

**Keywords:** adaptive learning, fog computing, fresh fruits and vegetables, internet of things, machine learning, supply chain, temperature break detection, temperature break prediction

## Abstract

**Introduction:**

Globally, 1.3 billion tons of food is lost or wasted each year, negatively impacting food security, the economy, and the climate. Fresh fruits and vegetables (FFVs), with their short shelf life and temperature sensitivity, are the most affected. This study systematically evaluates the integration of Machine Learning (ML), Adaptive Learning (AL), the Internet of Things (IoT), and Fog computing for temperature-break detection and prediction in FFVs supply chains. It critically evaluates their individual and combined capabilities, identifying compounding barriers that prevent genuine real-time integration of these technologies, while assessing their performance and operational readiness for real-time cold chain monitoring. Additionally, the role of fog computing in enabling efficient ML/AL deployment at the network edge is investigated for real-time applications in dynamic environments, with an implementation framework provided.

**Methods:**

Based on the PRISMA framework, searches of Scopus, Web of Science, IEEE Xplore, ACM Digital, supplemented by citation and reference chasing, produced 830 pre?deduplication records, identifying 14 relevant studies.

**Results:**

From the 12 analysed unique-dataset studies, 7 (58.3%) collected data using Basic Sensors, 3 with WSN (25%), and 2 with IoT (16.7%). Of the 14 ML studies, 5 (35.7%) detected temperature breaks, 5 (35.7%) predicted FFVs' temperature values, 3 (21.4%) predicted internal temperature (IT) values of a cold room or container, and 1 predicted IT values and time-to-temperature breaks. None of the studies predicted temperature breaks (event occurrence) or even their causes, while a few detected these breaks and their predefined causes. Four (28.6%) studies used IoT data, but none enabled live ML inference. None of the reviewed studies includes Fog or AL.

**Discussion:**

An integrated IoT-Fog-AL framework is thus proposed to address these gaps. These areas require focus to proactively reduce temperature breaks, thereby minimising food wastage and its associated effects, while also enhancing supply chain resilience and food security.

## 1 Introduction

Globally, one-third of food produced for human consumption (about 1.3 billion tons) is lost or wasted each year (WWF, 2017; Bai et al., 2023; Goedhals-Gerber et al., 2024). This food loss and waste not only exacerbates food insecurity but also squanders land, water, and energy and generates greenhouse gases that contribute to climate change (WWF, 2017; Fourie et al., 2023; Ndraha et al., 2018). Despite being nutrient-rich, fresh fruits and vegetables (FFVs) account for much wastage (Badia-Melis et al., 2018), especially in South Africa (44%), driven by post-harvest to retail supply chain phases (WWF, 2017; Goedhals-Gerber et al., 2024; Fourie et al., 2023; Ndraha et al., 2018). This underscores the need for robust cold-chain management to reduce food loss and waste, which is crucial for lowering production costs, improving food security and nutrition, and reducing greenhouse gas emissions (FAO, IFAD, UNICEF, WFP and WHO, 2022). Hence, the focus of this study is on the FFVs supply chain. Cold-chain failures, resulting from inadequate pre-cooling, storage, and refrigerated transport, are the primary cause of spoilage, as even brief temperature deviations can accelerate enzyme activity and microbial growth. Therefore, ensuring end-to-end temperature control preserves FFVs' quality and safety, extends shelf life, and supports Sustainable Development Goals on food security, reduced post-harvest loss, resource efficiency, and climate action (Badia-Melis et al., 2018; Bai et al., 2023; FAO, 2019; Findlay and Combrink, 2013; Goedhals-Gerber and Khumalo, 2020).

While the Internet of Things (IoT) has been used to monitor the cold chain in real time, it lacks predictive capability (Badia-Melis et al., 2018; Vilas-Boas et al., 2023; Zhong et al., 2023). This implies that IoT-based monitoring is reactive, detecting temperature breaks based on thresholds only after quality loss has occurred. Temperature breaks (or cold breaks) are defined as a scenario where product temperature deviates from the specified safe range for a given cold chain phase, potentially causing quality loss or safety risks (Meng et al., 2024). The integration of Machine Learning (ML) with IoT data unlocks transformative capabilities by automating processes, predicting outcomes, and enhancing real-time decision-making (Tzounis et al., 2017). Despite the predictive capability of ML, key challenges remain. First, models become outdated as new cold chain scenarios emerge, necessitating adaptive learning (Carnero et al., 2024; Guo M. F. et al., 2024). Secondly, receiving predictions before a break occurs is essential for proactive management. This can be addressed by fog computing, which brings computing (model deployment) closer to the data source, reducing latency (Musa and Vidyasankar, 2017; Ma et al., 2019; Solutions, 2015) and supporting real-time decision making (Solutions, 2015). Addressing these challenges, therefore, requires more than any single technology. Therefore, effectively monitoring temperature, detecting and predicting breaks in real time is beyond the capability of a single solution or technology. It requires the integration of multiple technologies, IoT, fog computing, ML, and adaptive learning (AL), into a complementary mechanism. Individually, each has limited impact and functionality challenges, but together they transform data collection, sharing, and analysis, and may be instrumental in reducing food loss and waste and associated impacts while increasing food security (Badia-Melis et al., 2018; Vilas-Boas et al., 2023; Zhong et al., 2023). This review, therefore, examines how integrating these technologies can strengthen the FFV's cold chain and reduce the immense global and South African burden of food loss and waste and their negative impact.

Several literature reviews have examined temperature monitoring and other parameter measurements in the FFVs cold chain using stand-alone sensors (basic sensors) (Vernier et al., 2021; Loisel et al., 2021; Yu et al., 2024; Badia-Melis et al., 2018) and Wireless Sensor Networks (WSNs) (Badia-Melis et al., 2018; Zhong et al., 2023). Although basic sensors (BS) and WSNs form the backbone of IoT implementations, they are not IoT; thus, these studies did not address IoT applications. One review reported on the integration of IoT with WSN (Qiao et al., 2024), and another on the use of BS in an IoT context (Onwude et al., 2020); meanwhile, the use of IoT for temperature monitoring in the FFVs cold chain was also discussed by Tzounis et al. (2017). However, among these studies, only (Tzounis et al., 2017) addressed the incorporation of ML for temperature prediction as well as IoT, and even then, the integration of ML with IoT was not examined. Similarly, no reviewed study explored the integration of ML with BS (Loisel et al., 2021) or with WSNs (Badia-Melis et al., 2018). Despite growing interest in FFV cold chain management, existing reviews remain largely descriptive and examine technologies in isolation, cataloging hardware innovations, packaging technologies, and the theoretical potential of IoT in the cold chain (Onwude et al., 2020; Badia-Melis et al., 2018; Loisel et al., 2021). To the best of the authors' knowledge, no systematic review has comprehensively mapped the sensing technologies deployed and their measurement limitations, assessed ML model performance for temperature break detection (TBD) and prediction (TBP), evaluated IoT–ML integration, or identified the barriers preventing real-time proactive break management. Notably, Loisel et al. (2021) flagged the absence of ML for TBD and TBP as a critical gap in 2021, yet no subsequent review has mapped the field's response, to the best of the authors' knowledge. Furthermore, to the best of the authors' knowledge, no study reports any use of Fog computing or AL in this domain. To fill these gaps, this review critically deconstructs the operational and algorithmic bottlenecks of current implementations, exposing the illusion of data sufficiency and IoT-ML integration, geographical and cold chain phase and product dataset biases, as well as the isolated use of temperature prediction from temperature break detection and temperature break prediction. The study also reveals the lack of studies implementing temperature break cause predictions, fog computing and adaptive machine learning in the FFVs domain, based on the reviewed studies. In doing so, it transitions the literature from theoretical promises to a practical solution, proposing a unified IoT–Fog–AL framework for end-to-end proactive break management in FFVs (Ahmadzadeh et al., 2023; Badia-Melis et al., 2018). A PRISMA-based systematic review methodology (Page et al., 2021b) is employed to answer the following questions: (i) What data collection technologies have been deployed for temperature monitoring in FFV cold chains, and what are their measurement limitations? (ii) To what extent have ML models been applied for temperature break detection and prediction in FFV cold chains, and what is the state of their performance? (iii) What is the current state of IoT-ML integration for real-time temperature break management, and what barriers prevent fuller adoption? and (iv) What gaps exist in current approaches regarding Fog computing and Adaptive Learning, and how could an integrated IoT-Fog-ML architecture address them? To address these, the following objectives will be pursued: (i) to evaluate data collection technologies deployed for temperature monitoring in FFV cold chains and characterize their measurement limitations (ii) to assess the extent and performance of ML model applications for temperature break detection and prediction in FFV cold chains (iii) to analyse the current state of IoT-ML integration for real-time temperature break management and identify barriers preventing fuller adoption. (iv) to investigate existing gaps in Fog computing and Adaptive Learning adoption and propose an integrated IoT-Fog-AL architecture to address them.

This paper is organized as follows: Section 2 outlines the PRISMA-based methodology. Section 3 presents the results, and Section 4 provides a comprehensive discussion of the findings and introduces the IoT-Fog-AL framework. Section 5 includes the conclusions and future recommendations.

## 2 Methods

Studying the integration, architectures and technical implementations of IoT, ML, AL, and Fog, in the FFVs cold chain and for temperature break detection and prediction is key, as it was not done before, according to the authors' knowledge. A systematic review entails a rigorous search for relevant studies, the appraisal of these studies, and the synthesis of their findings, all guided by established review protocols (Grant and Booth, 2009). This study was executed sequentially, premised on the Preferred Reporting Items for Systematic Reviews and Meta-Analyses (PRISMA) framework. In line with the systematic review, PRISMA has definite steps for systematically searching, identifying, and selecting articles, reviewing, synthesizing, and reporting (Moher et al., 2009; Grant and Booth, 2009). PRISMA was later on updated (Page et al., 2021b) and further expagorated (Page et al., 2021a). Covidence website tool was used for the PRISMA-based systematic review. Table 1 includes definitions of keywords which are instrumental in this study.

**Table 1:** Key definitions used in this study.

Construct	Definition
Real-time	The ability of a system to process data and produce an output within a latency that meets the operational deadline of the specific task, for instance, less than the sampling interval or the time required for effective intervention.
Temperature or cold breaks	Deviation of product temperature outside the specified safe range for a given cold chain phase, potentially causing temperature abuse, quality loss or safety risk (Meng et al., 2024; Swain and Jenamani, 2023a).
Temperature prediction	Forecasting a future temperature value (numerical)
Temperature break detection (TBD)	Identifying ongoing temperature break.
Temperature break cause detection	Identifying the causes of an ongoing temperature break.
Temperature break prediction (TBP)	Forecasting likely future temperature breaks (event occurrences).
Temperature break cause prediction	Forecasting the causes of future temperature breaks.
Real-time inference	A system where the ML model processes streaming sensor data as it arrives and generates predictions without significant delay (e.g., within seconds to minutes), allowing timely corrective action.
IoT-ML integration	A system where a trained ML model receives data from IoT sensors during active inference via continuous streaming. This inherently captures real-time capability (data flow is live).
Deployment readiness	The trained model is deployed and active during a live cold chain process (edge or cloud-based).

### 2.1 Eligibility criteria

The population, intervention, comparison, outcome and study design (PICOS) were used for the search strategy, as well as the inclusion and exclusion criteria (Methley et al., 2014) as shown in Table 2. It is an extension of the PICO (Methley et al., 2014; Page et al., 2021a). Studies involving cold-chain experiments or datasets labeled only as “perishables” or lacking a specified product type were excluded, retaining only those explicitly focused on fresh fruits and or vegetables to ensure clarity and avoid bias. Studies were included only where an ML model was trained and tested on data collected from FFV cold chain environments for temperature prediction, break detection, or break prediction. This includes studies employing basic sensors or WSNs, given the scarcity of literature on IoT-based temperature monitoring in FFV cold chains. This decision reflects the foundational role these technologies play in IoT ecosystems.

**Table 2:** The PICOS strategy used for search strategy, inclusion and exclusion criteria.

PICOS	Inclusion criteria	Exclusion criteria
Population	Fresh Fruits and Vegetables cold chains (storage, transport, distribution, and retail)	Non-FFV produce; studies not specifically involving FFV cold chain operations
Intervention	IoT as a data collection technology for training and testing ML temperature prediction (TP), TBD, and TBP models; ML for TP, TBD, and TBP; Fog computing and Adaptive Learning applied to FFV cold chain temperature management	Pure IoT monitoring studies without ML components; rule-based threshold detection without ML models; studies using Fog or AL in non-cold-chain contexts
Comparison	NA	NA
Outcome	Accuracy of ML models for TP, TBD, or TBP reported through quantitative performance metrics; IoT-ML integration level; real-time capability; deployment readiness	Studies reporting only qualitative outcomes without quantitative ML performance metrics; studies without evaluable results
Study designs	Primary, empirical research, including controlled experiments and case studies, implementing or evaluating ML models for TP, TBD, or TBP, AL, and Fog in FFV cold chains; published 2015 to 2025; English language only.	Review papers, theoretical papers, simulation studies without empirical validation, conference abstracts without full methodology; studies published before 2015 or after 2025; non-English publications

### 2.2 Information sources and search strategy

Scopus, Web of Science (WoS), IEEE Xplore, and ACM Digital Library online databases were used to find studies in the literature which are potentially relevant. These interdisciplinary databases were chosen for their extensive coverage and top-notch scientific papers suitable for systematic review. They have broader journal coverage than other databases, including in the natural sciences and engineering. The searches were done using Boolean expressions and were centered on key topics for the study, including “IoT, ML, Fog computing, Adaptive learning, fresh fruits and vegetables, cold chain, temperature prediction, temperature break detection or prediction.” A similar search strategy was used for Scopus and WoS databases, which were last searched on 17 January 2025, using search queries shown in Table 3. The IEEE Xplore and ACM Digital Library searches were conducted in April 2026, but results were limited to January 2025 for consistency. The original search strings were adapted for ACM Digital Library and IEEE Xplore to address database-specific constraints and improve search precision, as shown in Table 4. Specifically, IEEE Xplore's wildcard limit of 10 per query required removal of redundant truncated terms while retaining full explicit variants. Additionally, Fog computing and IoT queries were expanded to include a broader range of cold storage synonyms not present in the original strings. Consistent with the PICOS population criterion, Fog computing and Adaptive Learning queries were refined to include fresh produce variants. The IoT query was further refined to encode the core inclusion criterion directly into the search string, restricting results to studies collecting sensor data specifically for ML-based temperature prediction, break detection, or break prediction, consistent with the eligibility criteria applied during manual screening of the original Scopus and WoS results. This refinement improved search precision while maintaining conceptual consistency with the original search strategy. All searches were conducted based on title, abstract, and author keywords. Studies from 2015 to 2025 were selected to capture a decade of research for richer analysis. The year 2015 is also key, showing the transition of Fog computing and Applied Machine Learning from theoretical investigation to standardized implementation. Cisco's foundational white paper (Solutions, 2015) and the subsequent development of the OpenFog Consortium in 2015 established the current reference architectures. Similarly, the release of TensorFlow (Abadi et al., 2016) in 2015 provided the first industrial-grade, open-source framework capable of deploying Deep Learning models across heterogeneous edge devices, making “Intelligent Fog” theoretically practical. Peer-reviewed articles, scientific books and book chapters, conference papers, special issue editorial materials, and institutional documents such as dissertations, theses, or technical papers authored and published in English were considered. Review articles were excluded from the systematic search as the focus was on papers which implemented IoT, ML, Fog, or AL in the FFVs cold chain. In addition to structured database searches, a set of literature review articles was used, which reported on the use of IoT and ML in the FFVs supply chain as seed sources for forward and backward citation chasing (seed review DOIs in Table 5). This was done to identify any potential papers that could have been missed during the initial searches. For each seed review, the reference list was retrieved using Scopus and Web of Science, and the set of citing articles via Scopus on 11 April 2025. Records retrieved from 2015 to 2025 were screened using the same inclusion/exclusion criteria. All citation and reference-chasing exports were de-duplicated against the main databases' search results before screening. Some duplicates were removed autonomously by Covidence and verified by the first author, and a few that Covidence missed were removed manually.

**Table 3:** Search topics for retrieving documents in Scopus and WoS.

Topic	Query
TBD & TBP	(“temperature prediction” OR “temperature forecasting” OR “temperature anomaly predict*” OR “temperature break detect*” OR “temperature anomaly detect*” OR “cold break detect*” OR “temperature predict*” OR “temperature forecast*” OR “temperature anomaly forecast*” OR “temperature deviation*” OR “temperature monitor*” OR “temperature control*” OR “temperature break*') AND (“machine learning” OR “deep learning” OR “artificial intelligence” OR “neural networks” OR “DNN” OR “ANN” OR “AI” OR “NN” OR “ML” OR “computational intelligence” OR “statistical learning” OR “predictive model*” OR “forecasting model*” OR “time series predict*”) AND (“supply chain” OR “cold chain” OR “logistics chain” OR “distribution network” OR “supply network” OR “food chain” OR “temperature-controlled chain” OR “cold storage” OR “refrigerated transport” OR “cold room” OR “cooling room”)
AL	(“incremental learning” OR “online learning” OR “streaming learning” OR “sequential learning” OR “continual learning” OR “data stream learning”) AND (“machine learning” OR “deep learning” OR “artificial intelligence” OR “neural networks” OR “DNN” OR “ANN” OR “AI” OR “NN” OR “ML” OR “computational intelligence” OR “statistical learning”) AND (“supply chain” OR “cold chain” OR “logistics chain” OR “distribution network” OR “supply network” OR “food chain” OR “temperature-controlled chain” OR “cold storage” OR “refrigerated transport” OR “cold chain transportation” OR “cold room” OR “cooling room”)
Fog	(“fog computing” OR “fog-based computing” OR “fog architecture” OR “fog infrastructure” OR “fog systems” OR “fog services” OR “fog applications” OR “fog data processing”) AND (“supply chain” OR “cold chain” OR “logistics chain” OR “distribution network”)
IoT	wsn OR “Wireless Sensor Network” OR sensor OR iot OR “Internet of Things” OR iiot OR “Industrial Internet of Things”) AND (“supply chain” OR “cold chain” OR “logistics chain” OR “distribution network”) AND (“fresh produce” OR “fresh fruits” OR “fresh vegetables” OR “fresh fruits and vegetables” OR “perishable produce” OR “fresh foods” OR “agricultural produce” OR “farm produce” OR “fresh crops” OR “fresh harvest” OR “fresh agricultural products”)

**Table 4:** Search topics for retrieving documents in ACM Digital Library and IEEE Xplore.

Topic	Query
TBD & TBP	(“temperature prediction” OR “temperature forecasting” OR “temperature anomaly predict*” OR “temperature break detect*” OR “temperature anomaly detect*” OR “cold break detection” OR “temperature predict*” OR “temperature forecast*” OR “temperature anomaly forecast*” OR “temperature deviation” OR “temperature monitoring” OR “temperature control” OR “temperature break*”) AND (“machine learning” OR “deep learning” OR “artificial intelligence” OR “neural networks” OR “DNN” OR “ANN” OR “AI” OR “NN” OR “ML” OR “computational intelligence” OR “statistical learning” OR “predictive model*” OR “forecasting model*” OR “time series predict*”) AND (“supply chain” OR “cold chain” OR “logistics chain” OR “distribution network” OR “supply network” OR “food chain” OR “temperature-controlled chain” OR “cold storage” OR “refrigerated transport” OR “cold room” OR “cooling room”)
AL	(“incremental learning” OR “online learning” OR “streaming learning” OR “sequential learning” OR “continual learning” OR “data stream learning”) AND (“machine learning” OR “deep learning” OR “artificial intelligence” OR “neural networks” OR “DNN” OR “ANN” OR “AI” OR “NN” OR “ML” OR “computational intelligence” OR “statistical learning”) AND (“supply chain” OR “cold chain” OR “logistics chain” OR “distribution network” OR “supply network” OR “food chain” OR “temperature-controlled chain” OR “cold storage” OR “refrigerated transport” OR “cold chain transportation” OR “cold room” OR “cooling room”) AND (“fresh produce” OR “fresh fruits” OR “fresh vegetables” OR “fresh fruits and vegetables” OR “perishable produce” OR “fresh foods” OR “agricultural produce” OR “farm produce” OR “fresh crops” OR “fresh harvest” OR “fresh agricultural products”)
Fog	(“fog computing” OR “fog-based computing” OR “fog architecture” OR “fog infrastructure” OR “fog systems” OR “fog services” OR “fog applications” OR “fog data processing”) AND (“supply chain” OR “cold chain” OR “logistics chain” OR “distribution network” OR “supply network” OR “food chain” OR “temperature-controlled chain” OR “cold storage” OR “refrigerated transport” OR “cold room” OR “cooling room”) AND (“fresh produce” OR “fresh fruits” OR “fresh vegetables” OR “fresh fruits and vegetables” OR “perishable produce” OR “fresh foods” OR “agricultural produce” OR “farm produce” OR “fresh crops” OR “fresh harvest” OR “fresh agricultural products”)
IoT	(wsn OR “Wireless Sensor Network” OR sensor OR iot OR “Internet of Things” OR iiot OR “Industrial Internet of Things”) AND (“supply chain” OR “cold chain” OR “logistics chain” OR “distribution network” OR “supply network” OR “food chain” OR “temperature-controlled chain” OR “cold storage” OR “refrigerated transport” OR “cold room” OR “cooling room”) AND (“fresh produce” OR “fresh fruits” OR “fresh vegetables” OR “fresh fruits and vegetables” OR “perishable produce” OR “fresh foods” OR “agricultural produce” OR “farm produce” OR “fresh crops” OR “fresh harvest” OR “fresh agricultural products”) AND (“temperature prediction” OR “temperature forecasting” OR “temperature anomaly prediction” OR “temperature break detect*” OR “temperature anomaly detect*” OR “cold break detect*” OR “temperature predict*” OR “temperature forecast*” OR “temperature anomaly forecast*” OR “temperature deviation*” OR “temperature monitoring” OR “temperature control” OR “temperature break*”) AND (“machine learning” OR “deep learning” OR “artificial intelligence” OR “neural networks” OR “DNN” OR “ANN” OR “AI” OR “NN” OR “ML” OR “computational intelligence” OR “statistical learning” OR “predictive model*” OR “forecasting model*” OR “time series prediction”)

**Table 5:** Seed review papers used for citation and reference chasing.

#	References	DOI
1	Vernier et al. (2021)	10.3390/su13126702
2	Loisel et al. (2021)	10.1016/j.tifs.2021.03.052
3	Yu et al. (2024)	10.1016/j.fpsl.2024.101323
4	Badia-Melis et al. (2018)	10.1016/j.foodcont.2017.11.022
5	Zhong et al. (2023)	10.1016/j.fbio.2023.103350
6	Qiao et al. (2024)	10.1016/j.fbio.2024.103671
7	Onwude et al. (2020)	10.3390/pr8111431
8	Tzounis et al. (2017)	10.1016/j.biosystemseng.2017.09.007

### 2.3 Screening and selection of studies (inclusion and exclusion criteria)

The review protocol was not registered before data collection, which is acknowledged as a limitation. The search strategy, inclusion criteria, and data extraction procedures were defined before screening and applied consistently throughout in accordance with PRISMA 2020 reporting guidelines. A single reviewer (first author) used Covidence systematic review software for title and abstract screening, full text review, and data extraction. The PICOS criteria were applied at each stage to assess relevance and eligibility. Studies implementing IoT for collecting data for ML-based TP, TBP, and TBD, ML for TP, TBP, and TBD, or Fog and AL in FFV cold chains were selected for full-text review. After full text review, only papers meeting all defined PICOS criteria were selected for data extraction. The eligibility criteria were piloted on an initial set of records to ensure consistent interpretation before full screening commenced. There was no second independent reviewer available, and inter-rater agreement was not calculated, which is acknowledged as a methodological limitation.

### 2.4 Data collection

A data collection template form was created on the Covidence platform. The fields of the form include study and author identification information, the type of cold chain and fresh produce, the characteristics of IoT, ML, Fog, AL, and the integration of any of these technologies, as well as the outcomes.

### 2.5 Data items and analysis of studies

The data extracted from the relevant studies for IoT includes data collection technology, sensor types, data transmission protocol, intervals and storage. The data shows the details of the experiments for data collection in the FFVs cold chain, which can serve as a guide in conducting such type of experiments. ML models, their purpose, data used, integrations, technical implementations and metrics data were extracted for ML usage in the cold chain, which may also guide the use of ML for temperature analysis in the cold chain of FFVs. Tables and charts were used for analyzing, visualizing, and interpreting the data.

### 2.6 Study risk of bias assessment

Each study was assessed using a custom tool that comprises five domains, selected based on guidance for ML model assessment and cold chain requirements: (1) Reproducibility (ML pipeline transparency), (2) Dataset representativeness, (3) Validation methodology, (4) Reporting completeness, and (5) IoT-ML integration. Reproducibility was rated Low if the paper provided enough detail (preprocessing, features, algorithm parameters, and training) to re-implement the model without guesswork; Moderate if one major component was missing; High if multiple components were missing. Dataset representativeness was rated Low for real operational cold chain conditions, Moderate for controlled laboratory cold chain with real product. Validation methodology was rated Low only if a held-out test set (train/test split) was used; otherwise High (unsupervised studies exempt). Reporting completeness was rated Low if all key performance metrics were clearly reported; otherwise High. IoT-ML integration was rated Low for streamed data with ML edge or fog or cloud deployment, and High for batch or offline predictions. The Overall risk was High if any of the Reproducibility, Validation methodology, or IoT-ML integration was High, prioritizing study trustworthiness and real-world applicability, in line with the review's objective of identifying deployable systems. The first author assessed each study on Covidence, and no automation tools were used.

## 3 Results

The results from the search are summarized in this section.

### 3.1 Literature search

**Figure 1:**
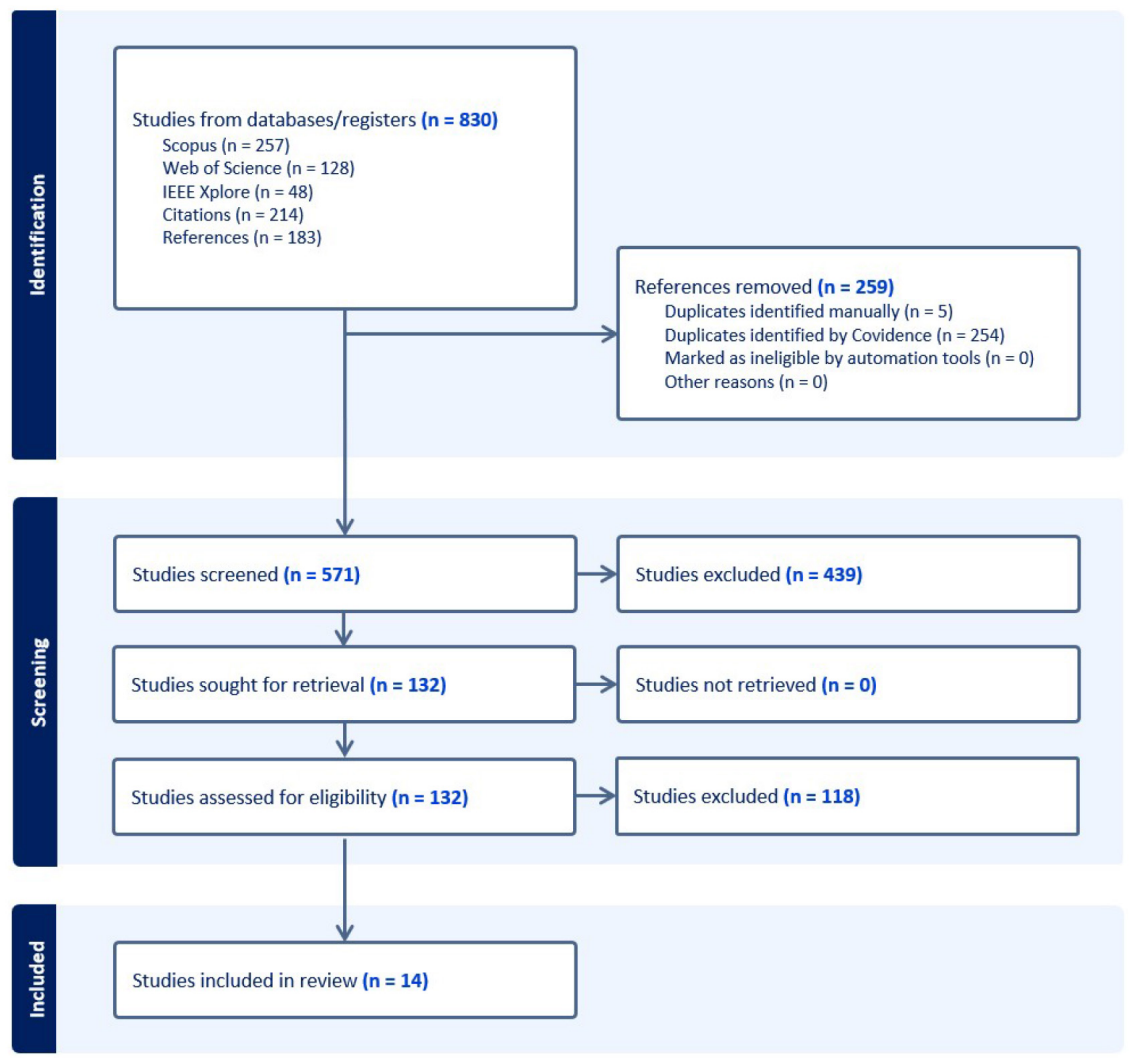
The PRISMA 2020 framework and flow diagram for the systematic review, incorporating searches from databases. Adapted from Page et al. (2021b,a). License: CC BY 4.0 (https://creativecommons.org/licenses/by/4.0/). Source: PRISMA 2020 statement/flow diagram template (https://www.prisma-statement.org/prisma-2020-flow-diagram).

Figure 1 shows the detailed information on the search and selection process. A total of 257 records were retrieved from Scopus, 128 from WoS, 48 from IEEE Xplore, and 0 from ACM Digital Library, based on the literature search; citation and reference searches yielded 214 and 183 potential studies, respectively. After removing 5 duplicates manually and 254 via Covidence, 571 unique studies remained for further analysis. During initial screening, 439 studies were irrelevant, whereas 132 full-text studies were assessed for eligibility. From the 132 studies, 118 were excluded after full-text assessment, leaving 14 studies whose findings are reported in the subsequent sections. None of the reviewed studies applied Fog Computing or Adaptive Learning in the cold chain management of FFVs, whether independently or in integration with IoT or ML systems.

### 3.2 IoT implementations in cold chain management

Before analyzing the specific operational and thematic constraints of the reviewed literature, it is necessary to outline the general IoT implementation landscape. Across the reviewed studies, in the cold chain of FFVs, few studies collected data using IoT and IoT with WSN and BS (Jiang et al., 2023; Swain and Jenamani, 2022, 2023a,b), as seen in Table 6. All the studies in Table 6 measured containers' internal temperature using different types of sensors, including ADT and DHT. Container's internal humidity was measured in some studies (Jiang et al., 2023; Swain and Jenamani, 2022, 2023a,b) using DHT sensors. Wi-Fi was used for data transmission (Jiang et al., 2023). Data was transmitted at 10-second (Swain and Jenamani, 2022, 2023a,b) and 10-min (Jiang et al., 2023) intervals. All the studies used the cloud for data storage and remote data access. Excluded from Table 6 are studies which used basic sensors (e.g., Meng et al., 2024; Loisel et al., 2022; Badia-Melis et al., 2016; Zou et al., 2023, 2025; Guo J. et al., 2024) and WSNs (e.g., Ayanoglu and Uysal, 2023; Xie et al., 2025; Haider et al., 2024) for data collection experiments in the FFVs cold chain but are part of the analysis in this section. Data collection technologies show significant operational constraints upon deeper analysis, which complicate their real-world usage.

**Table 6:** Comprehensive analysis of IoT implementations in cold chain management.

ID	Tech	Sensor types	Protocol	Interval	Storage	Source	References
1	IoT	IT (ADT7320), IH (DHT11)	ESP8266 & MQTT	10m	Ali cloud	Vegetable RT	Jiang et al., 2023
2	IoT	IT& IH (DHT22), CO_2_ (SCD30), VOC (MQ2)	?	10s	Cloud	Mangoes RT	Swain and Jenamani, 2022, 2023a,b

IT, Container's Internal Temperature; IH, Container's Internal Relative Humidity; RT, Refrigerated Transport; ?, Unclear.

### 3.3 ML implementations in cold chain management

Before analyzing the specific operational and thematic constraints of the reviewed literature, it is necessary to outline the general machine learning implementation landscape. Across the reviewed studies, as shown in Table 7, ML studies focus on temperature prediction, including virtual sensing, and temperature break detection in the FFVs cold chain. The number after # in the Sensor column provides an ID number linking it to an IoT-based experiment in Table 6. Various ML approaches were employed, including supervised learning (e.g., neural networks, ensemble methods, SVM, decision trees, and KNN) (Meng et al., 2024; Swain and Jenamani, 2023a; Zou et al., 2023), unsupervised learning (K-means), and anomaly detection (isolation forests, LOF) (Swain and Jenamani, 2022; Xie et al., 2025). The RMSE, MAE and Accuracy are the most prevalent metrics in descending order of usage for temperature-related regression and classification tasks as seen in Table 7 (Guo J. et al., 2024; Loisel et al., 2022; Badia-Melis et al., 2016; Jiang et al., 2023; Zou et al., 2023; Ayanoglu and Uysal, 2023). A 70% data split is predominantly used for model training, while the remaining is used for testing and or validation (Badia-Melis et al., 2016; Zou et al., 2023; Meng et al., 2024; Jiang et al., 2023), followed by 80% training data (Haider et al., 2024; Guo M. F. et al., 2024; Zou et al., 2025). The Adam optimiser is also the most used (Guo J. et al., 2024; Zou et al., 2023; Ayanoglu and Uysal, 2023; Jiang et al., 2023). While these standardized methodologies consistently yield high accuracy on isolated, study-specific datasets, a deeper thematic analysis reveals significant operational constraints that prevent their real-world integration.

**Table 7:** Taxonomy of 14 included studies: sensing, ML characteristics, and integration assessment.

Study	Country	Sensor	Product	CC phase	ML task	ML method	Metrics	Validation	RTP	DML	IoT-ML	Data
Meng et al. (2024)	China	BS	Banana	RT	TBD	3-layer BP-MLFFNN, DT & KNNs	Acc	Train/Val/Test split (70/15/15)	No	No	No	Exp
Xie et al. (2025)	China	WSN	Fruits & more	RT	TBD	iForests, SVM, LOF	P, R, F1, Auc	?	No	No	No	Op
Swain and Jenamani (2022)	India	IoT #2	Mango	RT	TBD + causes	K-Means	Silh, rand index	Clustering	No	No	No	Exp
Swain and Jenamani (2023a)	India	IoT #2	Mango	RT	TBD + causes	KNN, SVM, RF, LR, NB, perceptron	Acc	Train/Test split (70/30)	No	No	No	Exp
Swain and Jenamani (2023b)	India	IoT #2	Mango	RT	TBD + causes	Log R, SVM, Perceptron + FedAvg	Acc	Train/Test split (70/30)	No	No	No	Exp
Loisel et al. (2022)	France	BS	Apples	CS	PTP	3-5 layer-MLP with ReLU, RF, AdaBoost, LR, Lasso, SVM	RMSEs, RMSEt, RMSEi	Leave one-out	No	No	No	Exp+Syn
Badia-Melis et al. (2016)	Spain	BS & thermal	Apples	CS	PTP	3-layer MLP	RMSE, R^2^	Train/Val/Test split (70/15/15)	No	No	No	Exp
Zou et al. (2023)	China	BS	Citrus & banana	RT	PTP	MLFFNN, 3-layer LSTM, 2-layer BiLSTM	RMSE	Train/Val/Test split (70/15/15)	No	No	No	Exp
Zou et al. (2025)	China/Italy	BS	Strawberry, lychee, orange, apple	Pre-cool, CS, RT	PTP	2-layer BiLSTM + Digital twin	RMSE	Train/Test split (80/20)	No	No	No	Exp
Guo J. et al. (2024)	China	BS	Navel oranges	CS	TP & time-to-break	4-layer LSTM	RMSE, MAE	Train/Test split (80/20)	No	No	No	Exp
Emenike et al. (2016)	South Africa & Zambia	BS	FFVs	RT	Temp spatial estimation & ITP	NAR + NARX (ANN)	MSE	Train/Test split (70/15/15)	No	No	No	Op
Ayanoglu and Uysal (2023)	USA	WSN	Strawberry	RT	Temp spatial estimation	1D-CNN	MAE	Train/Val/Test split (60/20/20)	No	No	No	Op
Haider et al. (2024)	Pakistan	WSN	Tomato	RT	PTP	WOA-ELM, DT, LiM, NB, RF, SVM	P, R, and F1	Train/Test split (80/20)	No	No	No	Exp
Jiang et al. (2023)	China	IoT #1	Vegetables	RT	ITP & IHP	K-Means++ + LSTM & BP-MLP	RMSE, R^2^, MAE, MSE	Train/Test split (70/30)	No	No	No	Exp

Temp, Temperature; Pred, Prediction; PTP, Product Temp Pred; ITP, Container's Internal Temp Pred; IHP, Container's Internal Humidity Pred; ?, Unclear; RF, Random Forest; CNN, Convolutional Neural Network; SVM, Support Vector Machine; LR, Linear Regression; RMSEs, scenarios RMSE; RMSEt, over time RMSE; RMSEi, weight initialisation RMSE; BP, Backpropagation; MLFFNN, Multi-layer feedforward neural network; KNN, k-Nearest Neighbors; LiM, Linear Model; NB, NaBayes; WOA, Whale Optimization Algorithm; ELM, Extreme Learning Machine; NAR, Nonlinear AutoRegressive; NARX, NAR with Exogenous Variables; Log R, Logistic Regression; DT, Decision Trees; P, Precision; R, Recall; F1, F1-Measure; Silh, Silhouette; Acc, Accuracy; Exp, controlled laboratory experiment; CS, Cold Storage; RT, Refrigerated Transport; CC, Cold chain; Op, Data from real CC; Exp+Syn, experimental and synthetic data; RTP, Real-time Inference; DML, Deployed ML; IoT-ML, IoT-ML integration.

### 3.4 Quality appraisal and risk of bias assessment

Table 8 summarizes the quality appraisal and risk of bias assessment. Consistent methodological limitations emerged: dataset representativeness was predominantly experimental rather than operational, and real-time IoT-ML integration was absent in all studies, resulting in a “High” overall risk of bias. These patterns align with the field immaturity identified across the four thematic areas and confirm that the findings should be interpreted as proof-of-concept within specific experimental setups, with low generalizability to operational cold-chain systems.

**Table 8:** Quality appraisal and risk of bias assessment.

Study	Dataset	Validation	Reporting	IoT-ML	Reproducibility	Overall
Badia-Melis et al. (2016)	M	L	L	H	M	H
Emenike et al. (2016)	L	L	L	H	L	H
Loisel et al. (2022)	M	L	L	H	L	H
Swain and Jenamani (2022)	M	L	L	H	M	H
Ayanoglu and Uysal (2023)	L	L	L	H	L	H
Swain and Jenamani (2023a)	M	L	L	H	M	H
Swain and Jenamani (2023b)	M	L	L	H	M	H
Jiang et al. (2023)	M	L	L	H	M	H
Zou et al. (2023)	M	L	L	H	M	H
Meng et al. (2024)	M	L	L	H	M	H
Haider et al. (2024)	M	L	L	H	H	H
Guo J. et al. (2024)	M	L	L	H	L	H
Xie et al. (2025)	L	H	L	H	L	H
Zou et al. (2025)	M	L	L	H	L	H

L, Low risk of bias; M, Moderate risk; H, High risk; Dataset, dataset representativeness; Validation, validation methodology; Reporting, reporting completeness; IoT-ML, IoT-ML integration level; Overall, Overall risk.

### 3.5 Sensing the cold chain: from basic sensors to IoT and their measurement limitations

**Figure 2:**
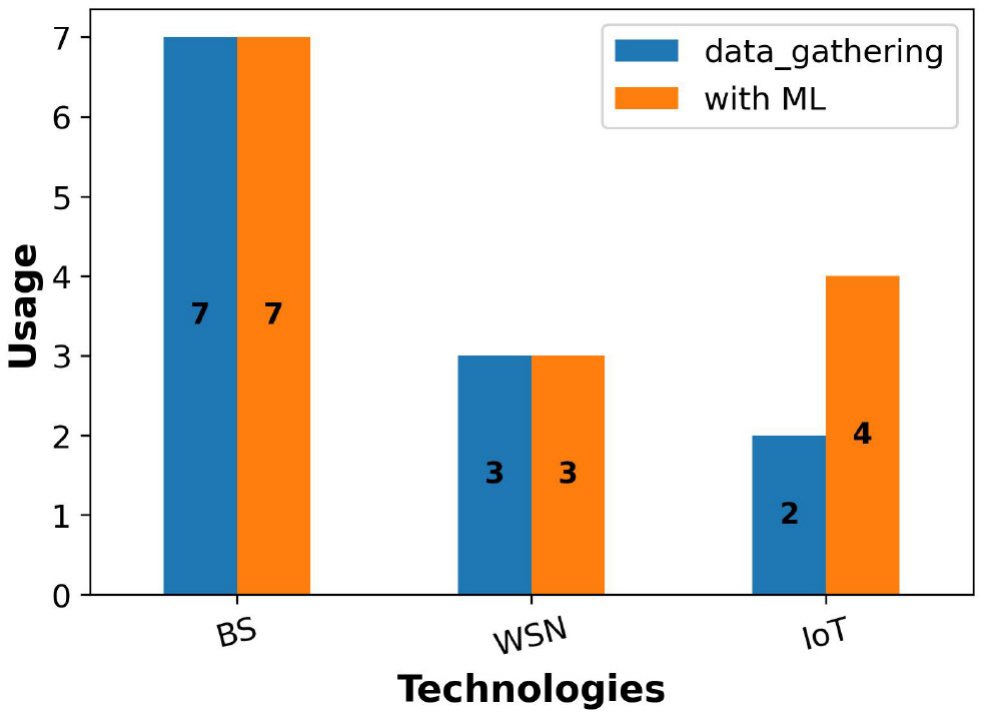
Data gathering technologies and ML integrations.

The foundation of modern cold chain monitoring has historically relied on basic temperature loggers and traditional tracking systems. Table 6 provides a detailed breakdown of the sensing technologies, protocols, data intervals, and storage approaches employed across the reviewed IoT implementations. As shown in Figure 2, 7 of the 12 studies using unique datasets (58.3%) used affordable basic sensors for data collection experiments in the FFVs cold chain (Guo J. et al., 2024; Loisel et al., 2022; Badia-Melis et al., 2016; Zou et al., 2023; Meng et al., 2024; Zou et al., 2025; Emenike et al., 2016). However, these conventional devices present a critical operational failure: they have limited storage, often require manual setup and data retrieval at the destination port, providing no real-time visibility or actionable information during transit. The devices have limited flexibility, hence the need for wireless sensors (Guo J. et al., 2024; Khumalo et al., 2023). To counter that, 25% of the studies (3) used WSN (Ayanoglu and Uysal, 2023; Haider et al., 2024; Xie et al., 2025), while very few (2, 16.7%) used IoT (Jiang et al., 2023; Swain and Jenamani, 2022, 2023a,b). Among the formally included IoT studies, data transmission employed Wi-Fi (Jiang et al., 2023), though connectivity challenges may be experienced in remote cold storage sites, even more for refrigerated trucks or shipments passing through poor connectivity sites. More broadly, IoT cold chain deployments have utilized GPRS, GSM, GPS, 3G, and 4G connectivity, though these technologies pose severe challenges within closed metal containers and during ocean transit where signal strength is limited (Khumalo et al., 2023; Wang et al., 2017). Furthermore, these networks are affected by the harsh deployment environment, with reading discrepancies in low-temperature or high-humidity levels affecting data reliability (Xie et al., 2025), confirming the findings of (Tzounis et al. 2017). Despite the progression toward active monitoring, profound physical and spatial measurement limitations persist.

The most prominent challenge to data quality is the extreme temperature heterogeneity within a densely packed refrigerated container, making sensor placement a critical determinant of system accuracy, as agreed by Badia-Melis et al. (2018) and Loisel et al. (2021). Optimizing sensor placement, such as placing sensors in the upper half of the pallet, following a diagonal mesh layout, or optimizing to downstream airflow, has been widely pursued (Ayanoglu and Uysal, 2023; Loisel et al., 2022; Meng et al., 2024; Zou et al., 2023; Khumalo et al., 2023; Swain and Jenamani, 2022, 2023a,b; Emenike et al., 2016). However, experimental evidence proves that placement alone drastically alters temperature monitoring accuracy (Vernier et al., 2021), which affects ML performances (Loisel et al., 2021). For instance, Loisel et al. (2022) demonstrated that a sensor placed at the back of a pallet yielded a Root Mean Square Error (RMSE) of 0.95^o^C, compared to 1.41^o^C when placed at the front. Because dense sensor deployment inside every fruit carton is economically prohibitive, data sparsity continues to affect ML model performance (Badia-Melis et al., 2016; Zou et al., 2023; Loisel et al., 2022; Jiang et al., 2023; Meng et al., 2024; Xie et al., 2025; Zou et al., 2025; Swain and Jenamani, 2022, 2023a,b). Systems are frequently forced to rely on single-source ambient data, but the quality of ML prediction is directly constrained by this limitation. For example, Meng et al. (2024) found that relying on a single ambient sensor resulted in a remarkably high false recognition rate of 97% for low-temperature alarms.

This spatial heterogeneity exacerbates the fundamental gap between ambient air temperature and the actual core temperature of the product, as confirmed in Badia-Melis et al. (2018) and Loisel et al. (2021). Accurately measuring the true internal temperature of fresh produce often requires conventional sensors with physical penetration probes; however, these methods are invasive and destructive to both the packaging and the product (Khumalo et al., 2023). Because dense, destructive sensor deployment is economically and practically infeasible, systems are forced to rely on ambient air data. Yet, the quality of ML model performance is directly constrained by this data source limitation. Zou et al. (2023) demonstrated that relying exclusively on a single ambient temperature sensor yielded a high RMSE of 2.35^o^C, proving that models trained solely on ambient data are inaccurate and unreliable proxies for actual product temperature. Thermal imaging has been explored as a non-destructive alternative, but it fundamentally fails to measure the interior of densely packed pallets and emissivity (Badia-Melis et al., 2016). Experimental evidence reveals this limitation causes massive discrepancies, ranging from 4^o^C to 12^o^C, between the recorded thermal surface temperature and the true internal core temperature of the product (Badia-Melis et al., 2016). Consequently, logistics organizers face a persistent bottleneck: a critical need to balance hardware costs, bandwidth requirements, and analysis accuracy without relying on dense, invasive sensor configurations or inaccurate ambient proxies (Zou et al., 2023; Meng et al., 2024).

To resolve these hardware bottlenecks and reduce deployment costs, ML is increasingly utilized for virtual sensing to predict temperatures at sensor-less locations. Because feature combinations dictate prediction accuracy, researchers are employing various optimisation strategies to identify the optimal minimum number of sensors required for reliable monitoring (Ayanoglu and Uysal, 2023; Zou et al., 2023; Meng et al., 2024). For instance, utilizing computationally inexpensive statistical methods like Pearson correlation and Dynamic Time Warping (DTW) to optimize sensor combinations, Ayanoglu and Uysal (2023) demonstrated that an ML model could use a single sensor to accurately predict temperatures at unmonitored locations with an error as low as 0.98°F, effectively matching the hardware accuracy of physical loggers. Beyond predicting temperatures at unmonitored locations, the same approach enables fault tolerance; when a physical sensor malfunctions or fails, ML can predict its expected readings from the remaining functioning sensors, maintaining system integrity without requiring physical sensor redundancy (Ayanoglu and Uysal, 2023).

These measurement limitations collectively mean the data foundation of cold chain monitoring is spatially compromised and intrinsically biased toward ambient rather than product temperature, undermining the reliability of every ML model trained on such data.

### 3.6 Detection vs. prediction: a field without a clear goal

**Figure 3:**
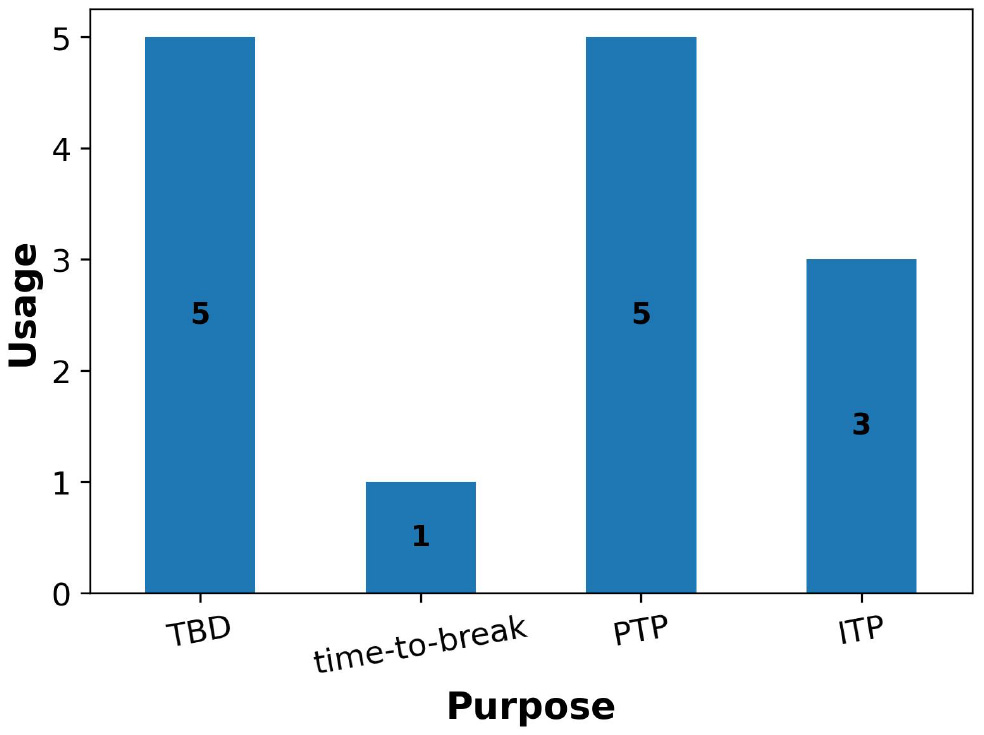
ML purposes.

Despite IoT enabling real-time data transmission, temperature break management in FFV cold chains remains fundamentally reactive. Even as machine learning increasingly permeates cold chain research, the literature reveals a fundamental conceptual gap: the absence of a unified framework distinguishing break detection (identifying ongoing abuse), break prediction (forecasting likely future breaks), cause identification (diagnosing the root nature of the detected break, such as a power failure or door opening), and cause prediction (forecasting causes of predicted breaks). Currently, studies treat these as isolated challenges rather than distinct analytical tasks that require different data structures, algorithms, and decision timelines. The gap was initially and partially cautioned in Loisel et al. (2021) for break detection and cause identification. To advance the field, as seen in Figure 3, 5 of the 14 ML studies (35.7%) detected temperature breaks (Meng et al., 2024; Xie et al., 2025; Swain and Jenamani, 2022, 2023a,b).

ML models demonstrate more accurate temperature predictions than traditional physics-based approaches such as Kriging interpolation and capacitor methods, bypassing the need for complex heat transfer modeling (Badia-Melis et al., 2016; Jiang et al., 2023; Loisel et al., 2022; Guo J. et al., 2024; Haider et al., 2024; Zou et al., 2025), which confirms existing studies (Loisel et al., 2021; Badia-Melis et al., 2018). While predictive models are becoming increasingly sophisticated, such as the utilization of BiLSTM networks to capture complex delayed thermal dependences inherent in packaged cold chains (Zou et al., 2025), these models still present a critical operational gap. Temperature value prediction, as shown in 8 (57.1%) of cases in Figure 3, is useful for shelf-life estimation, but it is insufficient for proactive break management because a predicted value alone does not determine break status or appropriate intervention and determining whether a predicted value constitutes a break still requires human interpretation and cold-chain expertise, introducing subjectivity and operator-dependent error. Though Guo J. et al. (2024) predicted threshold-crossing times with high temporal accuracy, they did not address break prediction as a discrete classification event, leaving their approach unvalidated for operational break prediction, where missed breaks carry severe consequences. Furthermore, their LSTM algorithm exhibited fundamental vulnerabilities in highly stabilized thermal environments, failing to predict the thermal curve of phase-change material transitions (R^2^ = 0.450). Because deep learning models over-learn prolonged periods of thermal stability, they struggle to forecast the sharp temperature spikes that follow, revealing a critical limitation in relying on standard time-series algorithms for proactive break forecasting. Additionally, if a model strictly predicts that a temperature break will occur, stakeholders are informed of the impending danger but are left entirely guessing as to the cause. Without knowing whether the predicted spike is due to a failing refrigeration unit, current temperature or humidity, operators lack the actionable intelligence needed to control and mitigate the risk proactively.

Conversely, the studies that do successfully classify pre-defined causes (power failures, door openings) of the breaks using IoT gathered data rely on reactive classification models (Swain and Jenamani, 2022, 2023a,b) or advanced anomaly filtering algorithms, such as improved Isolation Forests (iForest) that utilize cross-grouping strategies to escape the local optima traps of standard single-feature splitting (Xie et al., 2025). However, pre-defining the events or causes makes the models unable to handle new or undefined causes (Swain and Jenamani, 2022, 2023a,b), thereby excluding parameters such as past temperatures, O_2_, CO_2_, humidity, and many more. These diagnostic advancements remain completely isolated from predictive forecasting models, a critical limitation, as cause detection is inherently reactive. In contrast, cause prediction is an operationally valuable capability, as it enables intervention before the break occurs and reduces FFVs' qualitative and quantitative loss.

Despite the widely documented computational bottlenecks of deploying complex algorithms on edge devices, a critical void remains in the experimental literature. Among the studies analyzed, none successfully incorporated Fog computing or Adaptive Learning. Consequently, the literature fundamentally lacks empirical studies demonstrating their integration with IoT and machine learning, leaving the gap between localized sensing and real-time proactive control entirely unresolved. The field consequently cannot demonstrate cumulative progress; studies achieve high accuracy on their own controlled datasets, yet remain incomparable across datasets. Crucially, based on the current literature, no study has yet successfully integrated these capabilities to demonstrate proactive break forecasting, predicting both the impending temperature breaks and their specific root cause before the product experiences temperature abuse.

### 3.7 IoT-ML integration gap: why real-time systems remain elusive

IoT and ML complement each other: IoT provides real-time data streams, while ML provides predictive intelligence. Despite this, genuine integration between the two technologies remains rare in the reviewed literature for FFVs' cold chain temperature management. As shown in Figure 2, 7 (50%) of ML studies relied on basic sensors (Meng et al., 2024; Loisel et al., 2022), 3 (21.4%) integrated ML with WSN (Xie et al., 2025; Ayanoglu and Uysal, 2023), and only 4 (28.6%) integrated ML with IoT (Swain and Jenamani, 2022; Jiang et al., 2023). Moreover, of those IoT-ML studies, none demonstrated real-time temperature prediction. There is a big difference between studies that trained and tested ML models on static datasets, even if those datasets were originally collected by IoT sensors, and real-time deployment, where a pre-trained model continuously receives live sensor data as packets and makes predictions on the fly during active cold chain operations. Based on this definition, the reviewed studies do not show real-time IoT-ML integration because their models were trained and tested completely offline on datasets that had already been collected. This reveals a fundamental gap between data collection and intelligent real-time decision-making.

Connectivity poses significant challenges in cold chain environments (Khumalo et al., 2023). Although WiFi can be used for data transmission (Jiang et al., 2023), it is susceptible to signal interference and attenuation within metal reefer containers and large cold storage facilities due to electromagnetic interference from refrigeration equipment and signal absorption by dense product loads, since higher-frequency networks such as 2.4GHz exhibit poorer wave propagation inside closed metal containers compared to 433MHz (Wang et al., 2017). Though redundant sensor deployment may be a solution, it increases deployment costs (Zou et al., 2023; Badia-Melis et al., 2016). This becomes even more difficult when data has to be uploaded to centralized remote storage for ML analysis or storage, which may introduce latency and high bandwidth load (Swain and Jenamani, 2023b). To address this, edge-based analysis becomes an option, but edge-device computation remains challenging due to resourcing (Ribeiro Junior et al., 2022), especially for running ML models. With connectivity and edge-device capacity challenges in real-time systems, high data granularity strains resources (Zou et al., 2023); hence, the need to optimize data-collection intervals or reduce data-acquisition frequency (2–3 min) to optimize bandwidth and power use (Zou et al., 2023; Meng et al., 2024). Data privacy, isolation, and imbalance among containers or locations are also major challenges in IoT systems. However, Federated learning (FL) enables collaboration by sharing only model parameters rather than data, thereby addressing these issues and communication overheads (Swain and Jenamani, 2023b). However, FL also requires homogeneous model architectures as used in Swain and Jenamani (2023b).

Additionally, sensors are easily affected by a rugged deployment environment, including interference, which can be mitigated by insulating them with high-quality materials for protection (Ayanoglu and Uysal, 2023) and by predicting missing data from limited sensor measurements using ML (Jiang et al., 2023; Loisel et al., 2022; Ayanoglu and Uysal, 2023; Xie et al., 2025). Another major challenge is inferring detailed container temperature profiles from minimal sensor deployment with high accuracy; hence, the need to leverage the predictive power of advanced models such as BiLSTM (Zou et al., 2025). In situations where data are lost during collection due to network drops (Khumalo et al., 2023) or sensor malfunctions, researchers utilize methods ranging from linear interpolation (Jiang et al., 2023) to advanced ML estimation (Ayanoglu and Uysal, 2023) to reliably predict and repair incomplete data streams. Multi-source data are complex to analyse using ML (Jiang et al., 2023; Zou et al., 2023; Xie et al., 2025), and computational demands scale significantly with data dimensionality. Xie et al. (2025) found that their improved algorithm outperformed standard iForest significantly at higher dimensions with 30 sensor nodes, while performing comparably in low-dimensional conditions, demonstrating that algorithmic efficiency becomes increasingly critical as sensor networks grow. Data fusion using K-Means++ (Jiang et al., 2023) and data aggregation at a central sink node address data complexity, while a sliding window model stabilizes the variance inherent in continuous and noisy real-time sensor streams, though longer window length increases computational demands and accuracy (Xie et al., 2025).

These compounding barriers, namely connectivity failures, computational constraints, and data complexity, collectively explain why genuine real-time integration remains confined to a handful of experimental studies, leaving the field operationally immature despite its technical sophistication.

### 3.8 The data scarcity problem: a structural barrier to model generalization

Across the reviewed literature, the single most fundamental constraint limiting ML model development for FFV cold chain temperature break detection is the profound scarcity of accurately labeled real-world anomaly data. The scale of datasets underpinning the reviewed models reveals the depth of this problem. The largest labeled dataset identified contains only 400 annotated time-series samples across four event classes, generated from a single container experiment (Swain and Jenamani, 2023a,b). Furthermore, three of the reviewed ML studies (Swain and Jenamani, 2022, 2023a,b) were trained on data collected from this single IoT deployment, meaning that while they represent distinct model architectures and classification approaches, they do not represent independent datasets. The apparent diversity of ML studies, therefore, overstates the diversity of underlying data sources, compounding the dataset concentration problem.

At the same time, temperature prediction models were trained on as few as 10 cold chain scenarios (Loisel et al., 2022) and 62.95 h of historical data (Guo J. et al., 2024). Critically, no study in the reviewed literature utilized or contributed to a publicly available benchmark dataset. Because independent research teams are forced to conduct their own physical experiments and generate proprietary datasets from scratch (Meng et al., 2024), the training data inherently lacks diversity and fails to capture the operational complexity of real-world supply chains, rendering cross-study comparisons impossible and forcing each research group to restart the data collection process. It also forces researchers into a compromised trade-off between the high financial cost of deliberately sabotaging physical shipments and the inherent inaccuracy of relying on synthetic thermal models (Loisel et al., 2022). The data scarcity is also represented by the region, with more ML studies based in China (Meng et al., 2024; Zou et al., 2023; Xie et al., 2025; Jiang et al., 2023), with limited representation from India (Swain and Jenamani, 2023a), the United States of America (Ayanoglu and Uysal, 2023), Europe (Loisel et al., 2022), and Southern Africa (Emenike et al., 2016). It is also reflected in the cold chain phases covered, with more for transportation (Meng et al., 2024; Emenike et al., 2016; Swain and Jenamani, 2022, 2023b; Zou et al., 2023) and very few for cold storage (Loisel et al., 2022; Guo J. et al., 2024) and very few multi-phase coverage (Zou et al., 2025). Additionally, the scarcity extends to products, with mentioned and unmentioned multi-product studies leading (Zou et al., 2023, 2025; Xie et al., 2025; Jiang et al., 2023), followed by Mangoes (Swain and Jenamani, 2022, 2023a,b), with no single-product and cold room studies for Southern Africa.

To acquire genuine, annotated training data for supervised classification, researchers must resort to expensive physical experiments. For instance, Swain and Jenamani (2023a) were forced to deliberately induce temperature breaches by repeatedly turning off refrigeration power and leaving doors open for extended periods inside a 20-foot container loaded with 310 kg of mangoes to generate 400 labeled time-series samples (Swain and Jenamani, 2023b). Conversely, attempting to bypass these high experimental costs by relying heavily on synthetic data generated from physical thermal models introduces a fatal flaw. Loisel et al. (2022) demonstrated that models trained on experimental data performed 20% to 40% better than those trained on synthetic data. Because synthetic data are inherently noiseless, testing ML models on synthetic datasets leads to a substantial overestimation of their performance, by up to 150%, compared to their performance on real-world experimental data. Additionally, ANNs exhibit high prediction variance due to random weight initialization, which can be reduced by increasing the training data size (Loisel et al., 2022). Limited training and testing data affect ML models' performance (Emenike et al., 2016). Furthermore, ML models suffer from initial prediction uncertainty because product temperature data are unavailable at the start of a new cold chain journey (Loisel et al., 2022).

When researchers successfully capture real-world IoT data, the raw time-series streams are highly heterogeneous, requiring complex algorithmic pipelines to make them usable. First, a Cumulative Sum (CUSUM) change-detection algorithm must be deployed to detect mean temperature shifts and extract the relevant event-driven sub-sequences (Swain and Jenamani, 2023a,b). Because these extracted sequences have varying lengths, alignment algorithms such as Dynamic Time Warping (DTW) and Edit Distance on Real sequence (EDR) are required to warp and synchronize them. Finally, dimensionality-reduction techniques such as Dynamic Barycenter Averaging (DBA) are utilized to compute event centroids, thereby transforming the raw time-series into a compressed feature space for classification (Swain and Jenamani, 2023a). However, attempting to bypass the manual labeling process by using unsupervised clustering on these DTW distances often yields overlapping clusters and moderate accuracy, achieving silhouette scores as low as 62.5% (Swain and Jenamani, 2022).

To overcome the severe scarcity of high-quality labeled data without resorting to physical sabotage, the field is advancing toward distributed training architectures. Loisel et al. (2022) suggested the use of transfer learning to port learned features from previously documented tasks to new, data-scarce environments, reducing the demand for fresh experimental data. Expanding on this, FL frameworks are being utilized to draw value from highly imbalanced, scarce datasets across multiple containers. By utilizing Federated Averaging, individual edge nodes (reefer containers) train models locally on their specific, isolated break events and upload only the learned parameters to a central cloud server (Swain and Jenamani, 2023b). This creates a robust, generalized global model without requiring the centralized aggregation of massive, manually labeled raw datasets. Ultimately, the reliance on algorithmic workarounds such as transfer and FL highlights the field's core immaturity: researchers are forced to expend substantial computational effort to compensate for missing data rather than to uncover new thermal insights. Until the industry overcomes its reliance on isolated, proprietary experiments and establishes a standardized, open-access benchmark dataset, systems for cold chain temperature prediction, temperature break detection and prediction, and root cause identification and prediction will remain fragmented, localized, and fundamentally incapable of the generalization required for global, autonomous deployment.

## 4 Discussion

Despite advances in both IoT sensing and ML modeling, no study in the reviewed literature has demonstrated a system capable of simultaneously proactively predicting temperature breaks and their specific causes. This gap is not incidental; it reflects four compounding structural barriers that reinforce one another: a fundamental measurement compromise at the sensing layer, a systematic confusion of data sourcing with genuine real-time deployment, the absence of sufficiently labeled real-world training data, and the field's treatment of temperature and temperature break prediction and break cause detection as separate research streams, with proactive cause prediction remaining entirely unaddressed. Understanding why these barriers persist and how they interact is required before any effective architectural answer can be provided.

The consequences of this gap extend far beyond technical inefficiency. Approximately 44% of all FFVs are lost or wasted annually across the supply chain, resulting in billions of dollars in economic losses, including in South Africa (WWF, 2017). Where losses are passed on to consumers, FFVs become economically inaccessible to vulnerable populations, contributing to nutritional inequality (WWF, 2017). Reactive detection compounds these consequences: by the time a temperature break is identified, the product is already compromised and cannot be recovered, resulting in food insecurity (Goedhals-Gerber et al., 2024; WWF, 2017; Ndraha et al., 2018). While insurance mechanisms may compensate operators for financial losses, the spoiled product still decomposes, releasing greenhouse gases, permanently wasting the water, energy, and land resources consumed in its production (WWF, 2017) and reducing food safety (Ndraha et al., 2018). Proactive prediction of both the temperature break and its causes would allow operators to intervene before damage occurs, eliminating losses at the source rather than compensating for them after the fact, and enabling policy-makers to mandate evidence-based cold chain standards.

### 4.1 The measurement compromise

Addressing the identified gaps requires several foundational shifts in the field's approach to cold-chain ML research. To reduce FFVs' loss or wastage, it is paramount to conduct product temperature sensing rather than ambient temperature, as the two are different. ML models trained on ambient temperature data learn the association between air temperature and historical break patterns rather than the relationship between product core temperature and actual rotting risk (Zou et al., 2023; Emenike et al., 2016). Until product temperature sensing becomes economically viable at scale, or until ML models are explicitly trained to bridge the ambient-product gap, as demonstrated by Loisel et al. (2022) and Badia-Melis et al. (2016), the sensing layer will remain a fundamental constraint on model reliability regardless of algorithmic sophistication.

### 4.2 The conceptual separation of temperature and temperature break prediction

It is also important to not only predict a temperature value as done in reviewed studies (Loisel et al., 2022; Emenike et al., 2016; Zou et al., 2023; Guo J. et al., 2024), but also the status of the cold chain, as well as to evaluate the ML models for the classification task (Loisel et al., 2021). Though predicted values may be useful to an experienced cold chain operator, subjecting the “break” or “no break” judgement to an operator leaves room for errors of judgement, which depend on cold chain management experience, which is not universal. Good regression performance does not necessarily imply good break classification performance. A regression-optimized model that minimizes prediction error may nonetheless produce predictions that straddle permissible temperature thresholds (based on FFV cold chain phase) inconsistently, resulting in incorrect inference of break status. The model and evaluation metric chosen must consequently take into account how anticipated temperature values correspond with phase-specific allowable temperature ranges, rather than just minimizing numerical error. The reviewed literature does not explicitly address the distinction between regression accuracy and classification utility.

### 4.3 The data scarcity problem

Additionally, labeled cold-chain datasets must be publicly available to train and test ML models and enable investigations on temperature break cause predictions. Synthetic data overestimates temperature variance (Loisel et al., 2022), implying that models trained on simulated breaks develop a distribution that is not representative of reality. The external validity of controlled single-container experimental datasets cannot be established for real-world cold chains as well as heterogeneous commercial loads operating across varied routes and climate zones, and models derived from such data are inherently limited to recognizing the temperature breaks represented in training (Swain and Jenamani, 2022, 2023a,b). This is confirmed by the quality appraisal (Table 8), where most of the studies had a moderate risk of bias for dataset representativeness (Badia-Melis et al., 2016; Loisel et al., 2022; Swain and Jenamani, 2022). This reinforces the need to interpret the reported performance metrics with caution and highlights the immaturity of the field. The limited relevant global studies (14) from 4 databases, even covering 4 tasks, of which 9 virtually sense or predict temperature (Loisel et al., 2022; Badia-Melis et al., 2016; Guo J. et al., 2024; Ayanoglu and Uysal, 2023) for the FFVs cold chain, signal an immature field and niche, requiring much focus as FFVs contribute more to food loss and waste and associated impacts, due to their sensitivity to temperature fluctuations and high perishability (WWF, 2017; Goedhals-Gerber et al., 2024). The limited focus can be attributed to dataset scarcity, as the studies used 11 unique datasets, yet ML models require data for training and evaluation before deployment.

#### 4.3.1 Adaptive learning as a partial solution

Cold chain ML models, often trained on small, static, privately collected datasets (Loisel et al., 2022; Guo J. et al., 2024; Swain and Jenamani, 2023a), as reported in Section 3, Theme 4, and discussed earlier, cannot capture the non-stationary nature of real operational environments. Deploying such models without adaptation risks inaccurate predictions and consequent FFV loss or waste. The absence of AL in the reviewed literature does not diminish its importance; rather, it reflects the field's failure to address the non-stationary character of FFV cold chains. Many so-called static ML models are retrained periodically (e.g., weekly or monthly) using newly accumulated data. The limitation is not that they assume the world never changes, but that the retraining frequency is often too low to respond to rapid distributional shifts (e.g., seasonal changes, new routes), and the process is typically manual rather than automated. This leaves the model outdated between retraining cycles, as noted in analogous dynamic environments (Carnero et al., 2024; Aguilar-Palacios et al., 2019). Under the adaptive learning paradigm, models can be updated continuously or in small batches, reducing the lag between environmental change and model adaptation. Moreover, AL techniques also enable effective training even when initial labeled data are scarce, which is common in cold chain applications. Despite these advantages, no reviewed FFV cold chain study implemented AL, leaving a critical gap for real-world deployment. The feasibility of online learning for time-series forecasting in non-stationary environments has been demonstrated in IoT data streams (Carnero et al., 2024), while incremental learning with data replay effectively handles rare event detection under distributional shift (Guo M. F. et al., 2024). Until the industry commits to the systematic collection, labeling, and sharing of operational cold chain data across various goods, routes, and events, most models in this literature will remain an academic exercise, with operational validity assumed rather than demonstrated.

#### 4.3.2 Causal discovery as a response to label scarcity: temperature break cause prediction

Where labeled break data cannot be obtained through controlled experiments, time series lagged causal discovery methods such as Peter & Clark Momentary Conditional Independence (PCMCI) and Latent Peter & Clark Momentary Conditional Independence (LPCMCI), which are based on the combination of Peter & Clark algorithm (Spirtes et al., 2000) and momentary conditional independence (MCI) approaches offer a promising alternative, as they have demonstrated the capacity to infer causal relationships in analogous multivariate time series contexts, suggesting potential applicability for linking temperature breaks to ambient parameters such as humidity, CO_2_, and door status without requiring pre-labeled event data (Runge et al., 2019a, 2023, 2019b). Though temperature break detection and cause identification were encouraged in Loisel et al. (2021), this study further encourages predicting the would-be causes of a predicted break, which is very important. Without it, stakeholders may be informed of a pending break and may control the wrong parameter due to incorrect guesses, resulting in more future breaks and FFVs' loss or wastage. However, temperature breaks and cause prediction empowers cold chain stakeholders by informing them of the pending break, why it will happen, and how it can be avoided (countermeasures), enabling proactive and informed decision-making to reduce FFV wastage. Further to that, integrating temperature break prediction with temperature break cause detection, even with cause prediction, is also encouraged. If a break is predicted for the next hour, and operators are informed of the cause, which they control, it becomes important once that hour arrives to check if the break has been successfully avoided or its severity has been reduced.

#### 4.3.3 Geographical, cold chain phase, and product concentration

Geographically, the experimental data used to train and test these ML models are highly concentrated in China (Meng et al., 2024; Xie et al., 2025; Zou et al., 2023; Guo J. et al., 2024). The geographical bias implies that current models are optimized for specific regional climates and infrastructural conditions, potentially limiting their generalizability to global, cross-equatorial supply chains. A significant gap in the reviewed literature is the widespread reliance on single-commodity experiments, primarily isolated to the transportation phase (Haider et al., 2024; Jiang et al., 2023; Ayanoglu and Uysal, 2023). A comprehensive fresh fruit and vegetable (FFV) cold chain encompasses a continuous sequence of distinct operational stages, typically including post-harvest processing, pre-cooling, cold storage, refrigerated transportation, and temporary storage for retail display (Wang et al., 2017; Zou et al., 2025) yet many machine learning applications primarily focus on predicting temperature or detecting anomalies during refrigerated transport, largely ignoring the critical quality losses that accumulate during pre-cooling, cold storage, and retail where significant loss or wastage occur (Bai et al., 2023; WWF, 2017). Furthermore, the predominance of bananas (Meng et al., 2024), Indian mangoes (Swain and Jenamani, 2022), or apples (Loisel et al., 2022) highlights the need for focusing on other fruits. Thermal dynamics and quality kinetics differ substantially across types of fresh produce (Zou et al., 2025; Yu et al., 2024). This means that a predictive model based only on the rising respiration rates of climacteric fruits like bananas can't be used to make predictions about highly perishable, non-climacteric goods like strawberries (Meng et al., 2024).

Moreover, the near-total absence of studies from major FFV-producing and exporting regions in sub-Saharan Africa [except one study (Emenike et al., 2016)] and South America (Fruit South Africa, 2024) represents a significant blind spot. Cold chains in these regions operate under fundamentally different infrastructural, climatic, and economic constraints, including unreliable electricity (Wright et al., 2024), limited network connectivity, and extreme ambient temperatures, constraints that are absent from current models trained in controlled Chinese or European laboratory settings. Moreover, the absence of ML studies focused on the cold room phase and a product-specific cold chain is a huge dilemma for sub-Saharan countries, especially South Africa, because the cold chain is responsible for huge FFVs loss or waste, with 20-44% lost or wasted during cold storage and distribution (Bai et al., 2023; WWF, 2017). This is even more important as South Africa is the leading Southern Hemisphere fruit exporter, with fruits contributing much income and job creation (Fruit South Africa, 2024). Notably, a 2016 study conducted in a Southern African context demonstrated the feasibility of virtual sensing and predicting container temperatures for FFV exports using neural network models trained on experimental sensor data (Emenike et al., 2016). Despite demonstrating promising results and explicitly identifying real-time RFID integration as a priority for future work, no subsequent study in the reviewed literature built on this foundation in the Southern African context. This nine-year absence of follow-up research confirms that the geographical gap identified in this review is not merely a consequence of global research concentration; it reflects a specific failure to develop and deploy ML-based cold chain intelligence in one of the world's major FFV exporting regions, where the need is most acute, and the application would have the most direct impact on food security and export revenue. Future research must prioritize data collection and model validation in these under-represented regions, products and cold chain phases, if cold chain ML solutions are to achieve genuine global applicability.

### 4.4 The integration illusion

The combination of IoT data gathering and ML may be the most serious misconception in the studied literature, as confirmed by the quality appraisal (Table 8), where all the studies had a high risk of bias for the IoT-ML integration level, resulting in a high overall risk. The studies that employed IoT infrastructure acquired sensor data during controlled tests, saved it, and then used it to train ML models offline. The IoT hardware was used as a sophisticated data logger, not a real-time intelligence platform. This distinction is crucial for operational deployment (Swain and Jenamani, 2022, 2023a,b; Jiang et al., 2023). A model evaluated on historical IoT data differs from a model that operates on live IoT streams in terms of architecture, computation, and operations. Unlike temporarily-split batch or offline inference, streamed predictions must contend with data lateness, incompleteness, and binding computational constraints imposed by real-time processing requirements. The field's failure to discern between these two paradigms has resulted in an illusion of integration progress that obscures the genuine status of operational preparedness. Real-time cold chain intelligence necessitates not only IoT data but also a deployed inference pipeline capable of processing incoming sensor streams with latency compatible with intervention windows, which are often minutes rather than hours.

Multiple sensors generate big data (a variety of data at high velocity and in large volume) (Tzounis et al., 2017; Solutions, 2015) from within and outside cold rooms or refrigerated containers. However, big data may exceed available upload bandwidth, as may be the case at remote sites where cold rooms may be situated or a refrigerated transport drive-through, particularly in developing countries. Uploading the data to the cloud for analysis may introduce latency or upload failures and substantial bandwidth, particularly for heterogeneous data, which are undesirable for real-time applications intended to trigger a reaction in real time, such as in the cold chain scenario. To address these challenges, data for upload needs to be reduced, and computation needs to be brought closer to the data source in a distributed manner to enable real-time data analysis and support real-time decision-making. Then, the filtered data are sent to the cloud for long-term storage and further analysis. This is possible using fog computing. Fog computing is a cloud computing extension to the network edge that supports low-latency, location-aware and mobility-requiring applications (Musa and Vidyasankar, 2017). It offers faster processing near the source, which is required for real-time applications, compared to cloud computing. When decision support is provided in real time, key decisions can be made in real time as well, thereby reducing FFV wastage (Musa and Vidyasankar, 2017; Ribeiro Junior et al., 2022; Tzounis et al., 2017; Ma et al., 2019; Solutions, 2015). Given the connectivity challenges facing IoT deployments, especially in developing countries, computational constraints and data complexity, the importance of integration of ML and IoT with fog computing linked to the cloud cannot be overemphasized, yet existing reviewed studies lack fog computing for FFVs' cold chain management.

### 4.5 Proposed IoT-Fog-adaptive learning framework

This section presents the IoT-Fog-AL Framework, a response to the barriers presented in Section 3 and discussed earlier in this Section.

#### 4.5.1 Perception layer: addressing the measurement compromise

**Figure 4:**
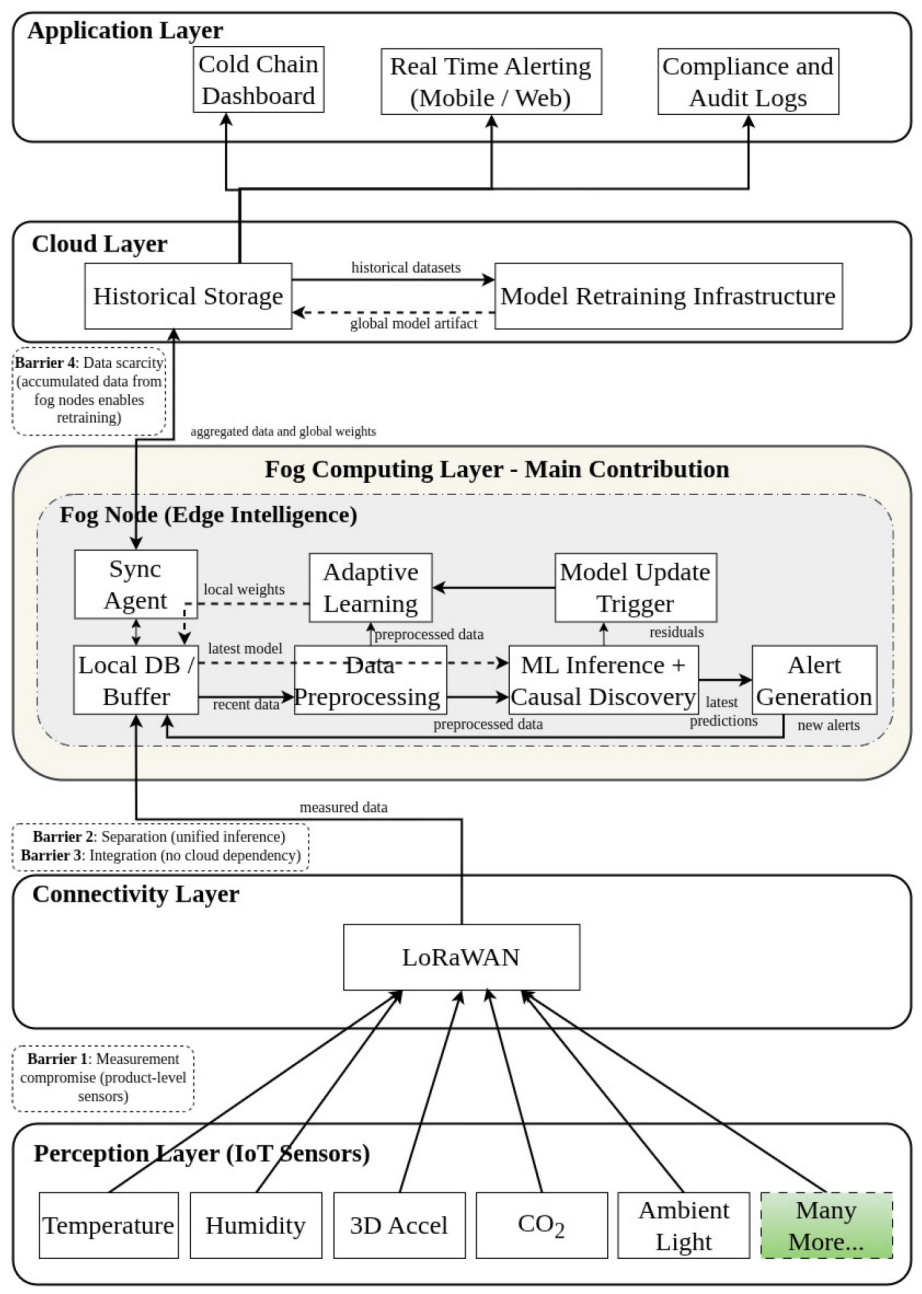
Proposed IoT-Fog-AL Architecture for the fresh produce cold chain adapted from Musa and Vidyasankar (2017). One-directional arrows indicate continuous, unidirectional data streaming, such as sensor data transmission to Fog Layer, while double-directional arrows represent bidirectional communication, such as state synchronization between Fog and Cloud Layers.

As shown in Figure 4, data is gathered by multiple sensors, such as temperature, CO_2_, O_2_, relative humidity, 3D-Acceleration, and ambient light within and outside FFVs' cold rooms or refrigerated containers, in the perception layer. These parameters, or features derived from them, are critical determinants of the accuracy of machine learning models used for temperature prediction in cold chain containers, especially in South Africa (Taguta et al., 2025, 2026). The ambient-product temperature difference indicated in Section 3, Theme 1, remains a structurally embedded obstacle that the framework recognizes but cannot fully address. Deploying product-level sensors on a commercial scale is economically prohibitive since sensors must be food-safe, calibrated per product type, and deployed across thousands of pallets per shipment. The framework trains ML models to estimate product temperature from strategically placed ambient sensors as an interim solution (Badia-Melis et al., 2016; Taguta et al., 2025). This minimizes measurement compromises through optimized sensor placement until product-level sensing becomes economically viable at scale. The raw telemetry is transmitted to the fog layer via the Network layer.

#### 4.5.2 Network layer: addressing connectivity barriers

The network layer addresses the connectivity issues raised in Section 3, Theme 3. The local area network (LAN) based on low-power, long-range, and highly penetrative (overcoming interference in humid food-dense cold chain deployments) LoRaWAN handles sensor-to-fog communication within containers, regardless of external network availability. LoRaWAN enables low packet loss during transmission (Kane et al., 2023; Bougaddou et al., 2024; Wang et al., 2017) and supports real-time and accurate predictions. It is suitable since this is textual data with a low payload.

#### 4.5.3 Fog computing layer: real-time edge intelligence

The payloads are decoded and published via MQ Telemetry Transport (MQTT) (Jiang et al., 2023) to the local message broker upon arrival at the Fog node, enabling an asynchronous “store-and-forward” architecture that separates data ingestion from computational processing. This ensures that no data is lost if the machine learning pipeline briefly overloads the edge device's CPU. The preprocessed data (including sequencing) is subjected to ML analysis using a local, predictive engine, comprising hybrid temperature-break detection, and prediction pre-trained ML models. When a temperature break is predicted, its cause can be discovered using LPCMCI (Runge et al., 2023), and a local alert is generated immediately, triggering SMS notification, dashboard update, or actuator response, without requiring cloud connectivity. Once the forecasted time window is reached, the detection model serves as a validation mechanism, confirming whether the early intervention effectively prevented the cold chain break. This makes the Fog layer the framework's key contribution, addressing the integration illusion highlighted in Section 3, Theme 3, and the collaborative temperature break detection and prediction with root cause prediction, as well.

When connectivity is available, the Sync Agent utilizes MQTT over secure TCP/IP to transmit aggregated data to the Cloud Layer via GSM, 4G, or WiFi (Jiang et al., 2023; Khumalo et al., 2023), with the fog node buffering all data locally during connectivity gaps. The data is aggregated to reduce bandwidth load and anonymized for safety (Solutions, 2015). Data synchronization takes place asynchronously to prevent inference latency and reliance on cloud availability. The feasibility of the proposed fog-based ML architecture is supported by a simulation study (Taguta et al., 2026) that deployed a deep neural network on a fog node for cold room temperature prediction. The fog node (simulated on a single-core CPU) achieved an average end-to-end latency of 84ms per prediction pipeline and an MAE of 1.3°C, significantly outperforming a cloud-based deployment (337ms, MAE 4.5°C). These results demonstrate that a fog node with modest computational resources can meet the latency requirements for real-time temperature break prediction. As a minimum hardware baseline for the fog node, the specifications successfully used for incremental learning in a related real-time detection task are adopted: a Raspberry Pi 4B with a 1.5GHz quad-core CPU and 8GB RAM (Guo M. F. et al., 2024). Fog nodes with comparable or greater resources can therefore support the proposed fog architecture.

Seasonal variations, changing product loads, route profiles, and equipment aging all cause distributional shifts that fixed models cannot accommodate. The Adaptive Learning module within the Fog layer addresses this by allowing fog node models to continuously update, independent of the cloud and internet, as new cold chain data accumulates, ensuring prediction reliability even under changing operational conditions and connectivity challenges. Drift detection algorithms separate true distributional shifts from transient noise before triggering updates, preventing model degradation due to unnecessary parameter changes (Zhang et al., 2024). This addresses the dataset scarcity in Section 3, Theme 4. IL and OL are two possible adaptive learning paradigms for the suggested fog architecture. IL updates the model in batches after accumulating fresh data (e.g., after a truck trip or a shift). It is well-suited to tasks where labeled instances occur infrequently, such as TBD, because confirmed breaks are rare. The OL model is constantly updated with new data points, making it ideal for real-time forecasting tasks such as TBP under gradual distributional variations. However, neither paradigm is solely focused on a single task; the choice is determined by hardware capacity, data availability, and latency requirements. IL is computationally feasible on a Raspberry Pi 4 (Guo M. F. et al., 2024), while true OL may require more powerful edge hardware (NVIDIA Jetson) to maintain below-100ms latency achieved in (Taguta et al., 2026).

Though adaptive ML (incremental and online learning) offers clear benefits for cold chain temperature break management, it also introduces specific risks that must be acknowledged. Online models can suffer from unstable updates when noisy or unrepresentative samples arrive, potentially degrading performance (Guo M. F. et al., 2024; Zhang et al., 2024). Concept drift mismanagement may occur if the drift detection threshold is poorly tuned, leading to either too frequent or too infrequent model updates (Zhang et al., 2024). Catastrophic forgetting, where the model overwrites previously learned knowledge when trained on new data, is a well-known issue in incremental learning (Carnero et al., 2024; Guo M. F. et al., 2024). False alarms may rise if the model over-adapts to rare events. Label scarcity is a critical challenge (Carnero et al., 2024), even for TBD, as confirmed temperature breaks may be rare in operational cold rooms and expensive to label, as they require break-inclusive experimentation (Swain and Jenamani, 2023a). Finally, poor performance under rare-event conditions is inherent to any data-driven model when positive examples are sparse, while computing resources are also a challenge (Carnero et al., 2024). The proposed framework mitigates some of these risks via drift detection (Zhang et al., 2024), data replay mechanisms (Guo M. F. et al., 2024), and periodic cloud-based resets. However, full robustness remains an open challenge for future work.

#### 4.5.4 Cloud layer: long-term data accumulation and model improvement

The Cloud layer solves Section 3, Theme 4's dataset shortage by collecting operational cold chain data, normal operation profiles, break events, break causes, and recovery sequences from all deployed fog nodes across several products, phases, routes, and climate zones. This aggregated dataset provides the operational labeled diversity that single-container controlled experiments cannot produce, gradually building the benchmark dataset infrastructure confirmed to be absent across all four databases searched in this review and reducing reliance on the synthetic data and transfer learning workarounds that the reviewed literature was forced to adopt. When enormous amounts of cold-chain-wide data accrue, the cloud infrastructure does heavy-batch retraining to create a globally optimal model. This revised baseline model is sent back down to the fog nodes, completing the adaptive feedback loop.

#### 4.5.5 Application layer

The historical data is visible in the Application layer. The layer provides stakeholders with cold chain remote monitoring via a dashboard and alerting. It also includes access to compliance and audit logs, which may be useful to settle disputes across cold chain operators, as well as promoting transparency and ease of compliance checks and mapping the cold breaks for future planning.

#### 4.5.6 Framework applicability

This architecture is specifically developed for South African local and cross-equatorial export scenarios: rural and remote packhouses and road connectivity are sporadic, and ocean travel eliminates cellular service, causing cloud-dependent systems to fail. The fog node's offline data processing and model adaptation capabilities, connected to sensors using SubGHz (LoRaWAN), are a fundamental deployment requirement for South Africa's local and global cold chains for apples, citrus and other fruits. This holds for packhouses located in poor-connectivity areas, as well as for consignments that travel through them (Ribeiro Junior et al., 2022; Musa and Vidyasankar, 2017; Wang et al., 2017). The travel may take three to six weeks via cross-equatorial sea routes to European markets, yet real-time temperature management remains important; otherwise, the products may be discarded if no corrective action is taken (Khumalo et al., 2023). Cloud-dependent architectures would fail during these inevitable periods of disconnection. To guarantee continuous inference and data buffering during grid disruptions, fog nodes in developing nations like South Africa must incorporate an uninterruptible power supply or battery backup due to load-shedding (Wright et al., 2024). LoRaWAN temperature sensors can operate on battery power for months to years, making them robust against load shedding (Bougaddou et al., 2024). The FFV's cold chain is a collection of phases, including pre-cooling, storage, transport, and retail, which include multiple stakeholders. It is thus important for such cold chain deployments to satisfy data governance and privacy rules, such as POPIA in South Africa and GDPR in Europe. The proposed IoT-Fog-AL framework promotes compliance by processing sensitive data locally at the fog node and sending only aggregated and anonymised summaries to the cloud (Solutions, 2015), ensuring that sensitive information is not exposed to other phases or non-responsible parties.

However, designing and developing such fog-based ML integrated and causally enabled systems is expensive, necessitating the utilization of simulation which are safe, cost-effective and reliable (Al-Hashimi et al., 2025; Taguta et al., 2026). Prominent generic simulators, including iFogSim, EdgeCloudSim, and FogNetSim++, rely on instruction abstraction, modeling applications as Directed Acyclic Graphs of abstract tasks defined by Millions of Instructions (MI) to evaluate architectures based on latency, throughput, energy consumption, and operational costs (Ghaseminya et al., 2025; Puliafito et al., 2020; Mahmud et al., 2022; Naas et al., 2018). Rather than executing actual logic, they use a mathematical reduction of these instructions over time based on a node's MIPS capacity (Shaik et al., 2022; Gupta et al., 2017). This makes them fundamentally incapable of running live ML inference as an active simulation task (Al-Hashimi et al., 2025; Scarpiniti et al., 2021). This points to the need for extending them or building custom ML-powered fog simulators which can serve as platforms to assess the infrastructure's feasibility and envisioned scenarios with the proposed framework before real-world deployment (Al-Hashimi et al., 2025; Scarpiniti et al., 2021; Taguta et al., 2026).

This study confirms that individual technologies (IoT, ML, Fog, and AL) have inherent weaknesses, but their complementary integration can overcome many of these limitations, resulting in a more capable system. For instance, ML models depend on quality data, which IoT can provide; conversely, ML can reduce the number of sensors needed for representative temperature mapping. The lack of security and big-data challenges associated with IoT can be addressed by Fog computing, while AL helps keep models up to date with recent data distributions. Until the FFVs cold chain community commits to partnering with research institutes for temperature break-inclusive cold chain data collection, and the researchers move beyond individual or separated IoT, Fog, or ML solutions as well as predicting temperature values without predicting the causes of any high or low temperatures to integrating the technologies, real time time decision making remains a mirage and FFVs among other fresh produce will continue to be lost resulting in food insecurity, economic losses, global warming and depletion of natural resources.

## 5 Conclusions and recommendations

This systematic review looked at four research questions. The review revealed that, despite advancements from basic sensors to IoT technologies, significant spatial, and measurement limitations remain, with temperature discrepancies in ambient products compromising the reliability of machine learning models. FFV's cold chain dataset scarcity also contributes to fewer studies for predictive temperature management, especially for sub-Saharan countries, where single-product and even cold storage ML studies are lacking. The review found that detection and prediction have been studied as separate areas of research when applying ML models. No study has shown a unified system that can simultaneously detect and predict temperature breaks and find the predicted break root causes. This is a major operational gap for proactive cold chain management. In the context of IoT-ML integration, due to connectivity challenges, limited computing power, and complex data, genuine real-time integration is lacking, though IoT have been used to gather datasets for offline ML studies. In the context of Fog computing and Adaptive Learning, no examined study utilized either technology within cold chain environments, indicating a significant architectural deficiency that an integrated IoT-Fog-AL framework could rectify. Thus, one has been proposed.

However, this review is subject to several limitations inherent to its scope and methodology. The exclusive use of Web of Science, Scopus, IEEE Xplore, and ACM Digital Library, combined with a search window of 2015 to 2025 and restriction to English-language publications, may have excluded relevant work published in other languages or indexed in other databases such as Google Scholar. The focus on FFVs specifically excludes findings from studies on other perishables, such as meat, dairy, and seafood, that may offer transferable insights. Additionally, primary authors were not contacted to clarify experimental conditions or dataset characteristics, meaning some methodological details may have been interpreted from reported findings alone. The absence of dual-independent screening (only one reviewer) may introduce bias. Future reviews will employ dual independent screening to enhance reliability. Future research may include studies before 2015, consider other literature databases and focus on other perishables such as meat, dairy, seafood, and flowers, among others.

The results of this study have direct implications for cold chain operators, ML researchers, IoT system designers, and food-safety policy-makers. They all point to the urgent need for open-access benchmark datasets, standardized evaluation frameworks, and integrated real-time architectures capable of three interconnected functions: predicting temperature breaks and their causes before they occur; triggering and supporting proactive interventions to avert the predicted break; and subsequently confirming, through break detection, whether those interventions were effective. Until the FFV cold chain community agrees to work with research institutes to collect cold chain data that includes breaks, and researchers stop working on separate IoT, Fog, or ML solutions and start integrating them, real-time decision-making will remain a dream. This will lead to more lost FFVs, causing food insecurity, economic losses, global warming, and the depletion of natural resources. Yet regulatory (POPIA, GDPR), intellectual property, and commercial barriers make such collaboration difficult, as they will not be solved by goodwill alone.

## Data Availability

The original contributions presented in the study are included in the article/supplementary material, further inquiries can be directed to the corresponding author.
